# A Case of Aquagenic Syringeal Acrokeratoderma in a Male Patient Undergoing Treatment With an Angiotensin-Converting Enzyme Inhibitor

**DOI:** 10.7759/cureus.74157

**Published:** 2024-11-21

**Authors:** Justin Lindsay, Aurelia R Incristi, Bryson Arnett, Marcelo Costa, Christian Chong

**Affiliations:** 1 Medicine, Wright State Boonshoft School of Medicine, Dayton, USA; 2 Internal Medicine, Kettering Health Network, Kettering, USA

**Keywords:** aquagenic palmoplantar keratoderma, aquagenic pruritus, aquagenic syringeal acrokeratoderma, aquagenic wrinkling, aquagenic wrinkling of palms

## Abstract

Aquagenic syringeal acrokeratoderma (ASA) is a rare dermatological condition characterized by the transient appearance of edematous, white, translucent papules on the palms following water exposure. While the condition is most commonly associated with cystic fibrosis (CF) and predominantly affects young women, this report presents a unique case in a 24-year-old man without a history of cystic fibrosis. The patient reported a 10-month history of painful, pruritic eruptions on the hands following exposure to water. Symptoms resolved within an hour post-exposure but were persistent and increasingly severe over time.

The patient's medical history was unremarkable, except for the use of lisinopril for hypertension and propranolol for performance anxiety. Given the absence of CF, the etiology of the disease in this patient remains unclear; however, the use of an angiotensin-converting enzyme (ACE) inhibitor is hypothesized to have contributed to the onset of symptoms through mechanical mechanisms involving sodium retention and osmotic gradient disruption in keratinocytes.

This case highlights the diverse clinical presentations and emphasizes the importance of considering ASA in the differential diagnosis of patients without CF or other traditional risk factors. This case underscores the need for further research to elucidate the underlying mechanisms and improve diagnostic accuracy for this rare but potentially debilitating condition.

## Introduction

Aquagenic syringeal acrokeratoderma (ASA) is an exceptionally rare acquired disease first described by English and McCollough as "transient reactive papulotranslucent acrokeratoderma" in 1996 [1]. ASA is characterized by edematous, white, translucent papules and plaques that transiently localize on the palms and, less commonly, the soles, after immersion in water for 3-5 minutes [2-4]. The reaction lasts between 20 and 30 minutes after removal from water immersion and drying [3,5]. Symptoms can also include tingling, pain, a sense of tightness, burning, or itching in the hands [2]. Diagnosis is typically clinical and involves the "hands-in-the-bucket" technique [1].

ASA most commonly presents in young women or those with cystic fibrosis (CF); other common etiologies include asthma, allergic rhinitis, urticaria, palmar erythema, and malignant melanoma [6]. This case report aims to illustrate an unusual presentation of ASA in a young adult man without a history of CF on an angiotensin-converting enzyme (ACE) inhibitor, contributing to the understanding and pathophysiology of this condition beyond its association with CF [1,2,4]. This underscores the potential underdiagnosis of ASA in populations not affected by cystic fibrosis.

## Case presentation

A 24-year-old male patient reported a 10-month history of white papular eruptions on both palms with burning, pruritus, and pain following exposure to hot water and when sweating in nitrile gloves for 5-10 minutes. The eruptions disappeared within 30-60 minutes after drying the palms. The patient used moisturizer following the initiation of events to help with symptoms, which provided no relief. The patient has no history of other skin conditions, drug use, or associated past trauma. Relevant pharmaceutical history includes lisinopril 10 mg by mouth daily and propranolol 60 mg as needed for performance anxiety.

**Figure 1:**
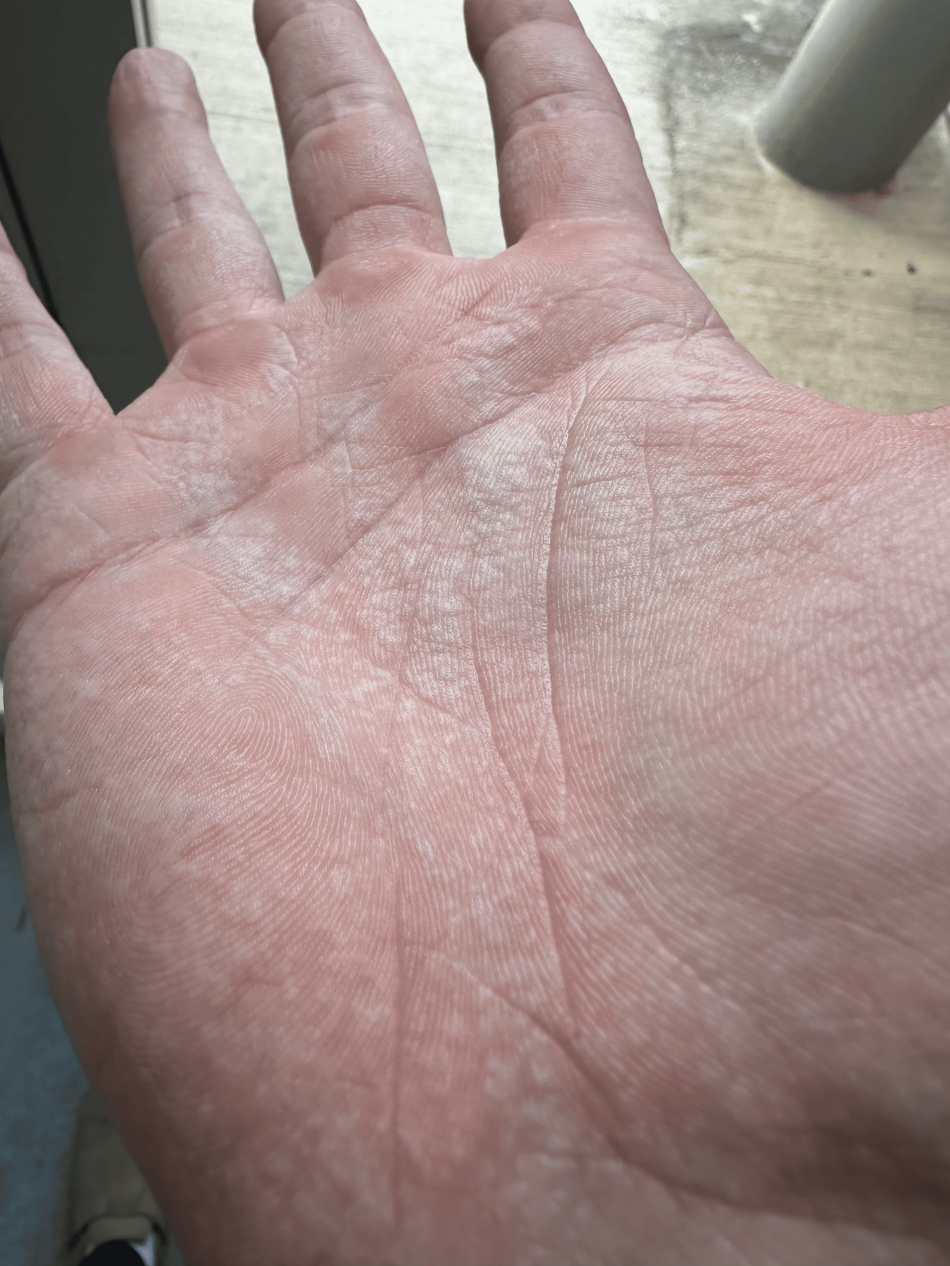
ASA presentation on the right palmar surface that occurred on August 14, 2024 Right palmar surface illustrating the transient appearance of edematous, white, translucent papules following extended exposure to water. Papules ascend up the metacarpals laterally. The figure also displays diffuse redness along a similar palmar pattern. ASA: aquagenic syringeal acrokeratoderma

The dermatological evaluation revealed that the following changes occurred upon contact with contained sweating or hot water: hypopigmentation in both palmer regions ascending the fingers, redness of the skin, increased palmar creases, and white shiny papules of variable sizing. Physical examinations were unremarkable. Due to the patient's clinical symptoms, a biopsy was not taken, and histopathology was not needed for diagnosis. Additionally, a water immersion test was not conducted because the patient had recurrent positive results upon submersion in water at home. This suggests an unknown etiology, and the patient was advised to avoid situations that incite the initiation of symptoms. The patient was given aluminum chloride hexahydrate (ACH) to aid in symptomatic relief. The initial event occurred in November 2023. As time progressed, the frequency of events increased, with the patient experiencing symptoms each time they had contact with water for longer than a few minutes in duration. In addition, severity also increased, as defined by the extent of palmar wrinkling, prominence of white, translucent papules following water exposure, the duration of symptoms after water contact, and the degree of discomfort or pain experienced. Currently, the patient continues to use ACH for symptom relief in addition to lifestyle modifications with the specific recommendation that the patient avoid prolonged exposure to water. This has led to improvement in the severity but not frequency of events. Clinical photographs taken on the initial date of presentation and the past seven months were collected and documented (Figure 1).

## Discussion

Previously, ASA was thought to be predominantly associated with CF, as CF has a vast spectrum of effects on multiple organ systems. It is thought that higher sodium concentrations from CF mediate increased water-binding capacity of keratins in the epidermis [7].

Potential pathogenic mechanisms include increased sodium concentration in the skin, which leads to increased water retention in the stratum corneum, structural or functional defects in the stratum corneum, dysregulation of aquaporin 3 channels, and the role of weakened eccrine duct walls [3].

The forenamed mechanisms can be explained histologically by the presence of orthohyperkeratosis with increased thickness and abnormal staining of the stratum corneum; dilated dermal eccrine ducts, as well as hyperplasia of eccrine glands; and increased capillaries around and adjacent to the eccrine glands [3,8]. This description is consistent with other reported cases describing compact orthokeratosis, hypergranulosis, and dilatation of intraepidermal eccrine ducts [9]. The dermis showed dilated blood vessels and numerous eccrine glands. While the presentation of ASA varies, it seems that there is a consistent histological display suggesting that despite variable inciting events or history, the mechanism remains the same in all those with ASA.

While there has been a strong association between ASA and CF, ASA has also been shown to be drug-induced independent of CF history [3,10]. Medication-induced ASA has been documented with cyclooxygenase-2 (COX-2) inhibitors, non-steroidal anti-inflammatory drugs (NSAIDs), gabapentin, ACE inhibitors, acetylcholinesterase enzyme inhibitors, aldosterone receptor blockers, and tobramycin [10,11]. These medications enhance sodium retention and the osmotic gradient within epithelial cells, leading to water uptake by the stratum corneum [10]. More specifically, it is hypothesized that the mechanism behind NSAID induction of ASA is that COX-2 inhibition in epidermal cells may cause increased sodium reabsorption similar to the effect of COX-2 inhibitors on kidney cells [3,7,12]. In the kidney, the inhibitory effects of NSAIDs on COX-2 increase sodium reabsorption. Similarly, COX-2 is expressed in the keratinocytes in hair follicles, sweat glands, and epidermis and is associated with keratinocyte differentiation [7,12]. Thus, the effects of NSAIDs on COX-2 expression in the skin may cause sodium retention in epidermal keratinocytes [10]. Sodium retention is also the main proposed mechanism behind the potential association of ACE inhibitors with ASA [10].

Given the past medical history and the age of the patient, it seems that aquagenic syringeal acrokeratoderma's traditional link to CF is an unlikely mechanism for the pathogenesis of this condition. This case report suggests that the patient's onset of ASA is following the initiation of the ACE inhibitor lisinopril, which has contributed to his present condition by potentiating sodium retention and/or disruption of an osmotic gradient present in keratinocytes [10].

This patient received ACH to decrease the overall hydration of the hands by blocking the sweat glands. Other available treatments include a 20% aluminum chloride solution, botulinum toxin injections, iontophoresis, antihistamines, pomade containing 5% salicylic acid, a mixture of mometasone furoate and petroleum jelly, and a cream containing 20% urea [6]. It is recommended that patients limit activities that elicit symptoms and avoid prolonged water immersion.

## Conclusions

Aquagenic syringeal acrokeratoderma (ASA) is a rare condition characterized by pruritic, edematous, translucent papules localized to the palms and occasionally the soles that leads to patient discomfort and can potentially alter quality of life. While previously thought to be highly associated with the female gender and CF, this case study illustrates the presentation of a young male patient without a history of CF who was recently started on an ACE inhibitor. This case presentation could potentially elucidate a new drug association between ASA and ACE inhibitors. This disease presents as a potentially debilitating condition for those who are unable to find a way to limit exposures eliciting symptoms. In this specific case, the condition interferes with the patient's future occupation. Clinicians should focus on symptom management and ensuring the patients' occupation and lifestyle are considered when initiating treatment.
